# Intraosseous migration of supraspinatus calcification: benefits of intraoperative ultrasound technique

**DOI:** 10.1016/j.xrrt.2023.09.012

**Published:** 2023-10-23

**Authors:** Victor Housset, Vincent Martinel

**Affiliations:** a Clinique Maussins Nollet - Ramsay Santé, Paris, France; b Groupe Orthopédie Ormeau Pyrénées, Polyclinique de l’Ormeau - ELSAN, Tarbes, France

**Keywords:** Calcifying tendinitis, Arthroscopic ultrasound-guided needling, Intraosseous migration of supraspinatus calcification, Ultrasound-guided surgery, Supraspinatus calcification, Rotator cuff calcification

Calcifying tendinopathy (CT) of the cuff remains a common condition and affects 2%-22% of the general population.^26^ The clinical course of this entity as described by Uhthoff^25^ is well known, with a final phase of shedding into the superficial bursa that is frequently extremely painful.^1^ The natural history of calcifying tendinitis can also be associated with endocrine disorders with an earlier onset of symptoms, a longer natural history and a significantly higher rate of patients who underwent operative treatment in case of associated endocrine disease.^7^ The predictive failure factors of conservative management for the treatment of shoulder calcifying tendinitis have previously been described including calcific lesions of more than 1 centimeter.^5^

Although perfectly described in the literature, intraosseous migration of calcium deposits remains a rare possibility, still relatively unknown, continuing to confuse many physicians, whether general practitioners, radiologists, sports physicians, or orthopedic surgeons. It has been previously demonstrated on both functional data and imaging that calcific tendinitis of the rotator cuff with tuberosity osteolysis was a distinctive form from classical calcific tendinitis and was associated with significantly lower Constant scores preoperatively and postoperatively.^20^

In this situation, magnetic resonance imaging (MRI) imaging, which is now frequently used, can lead to a wrong diagnosis, because it sometimes causes pseudotumoral images.^9^^,^^14^ In case of intraosseous migration, CTs can have an unusual appearance that can be confused with pseudotumoral calcinosis or secondary localization of neoplasia with bone tropism.^24^

Arthroscopic resection is a common procedure that may be indicated in CTs of the rotator cuff if conservative management (including physiotherapy, infiltration, or ultrasound-guided needling aspiration) have failed. It has been demonstrated that removal of the calcific deposit alone was a better surgical option than associated with subacromial decompression which required a longer time to return to unrestricted pain free activities.^15^

However traditional arthroscopy using the bursal view may be challenging and time-consuming due to difficulty locating the calcification. If it is difficult to clearly visualize the precise location of the CT, intraoperative fluoroscopic imaging may be necessary. The use of Arthroscopic ultrasound-guided needling (A-USGN) is an increasingly frequent procedure for the treatment of rotator cuff calcifications. This compact device has the advantage of locating the calcification rapidly and easily1^16^^,^^17^ and providing useful 3-dimensional images with nonirradiating, especially when the calcification involves small deposits, in the infraspinatus or the subscapularis tendons.

This complex case report illustrates the value of the surgeon performing ultrasound of the shoulder to diagnose an intraosseous CT. It also shows the interest of perioperative ultrasound-guided management in this form of the disease, which is more difficult than intratendinous forms.

## Case report

### Case presentation

**Figure 1:**
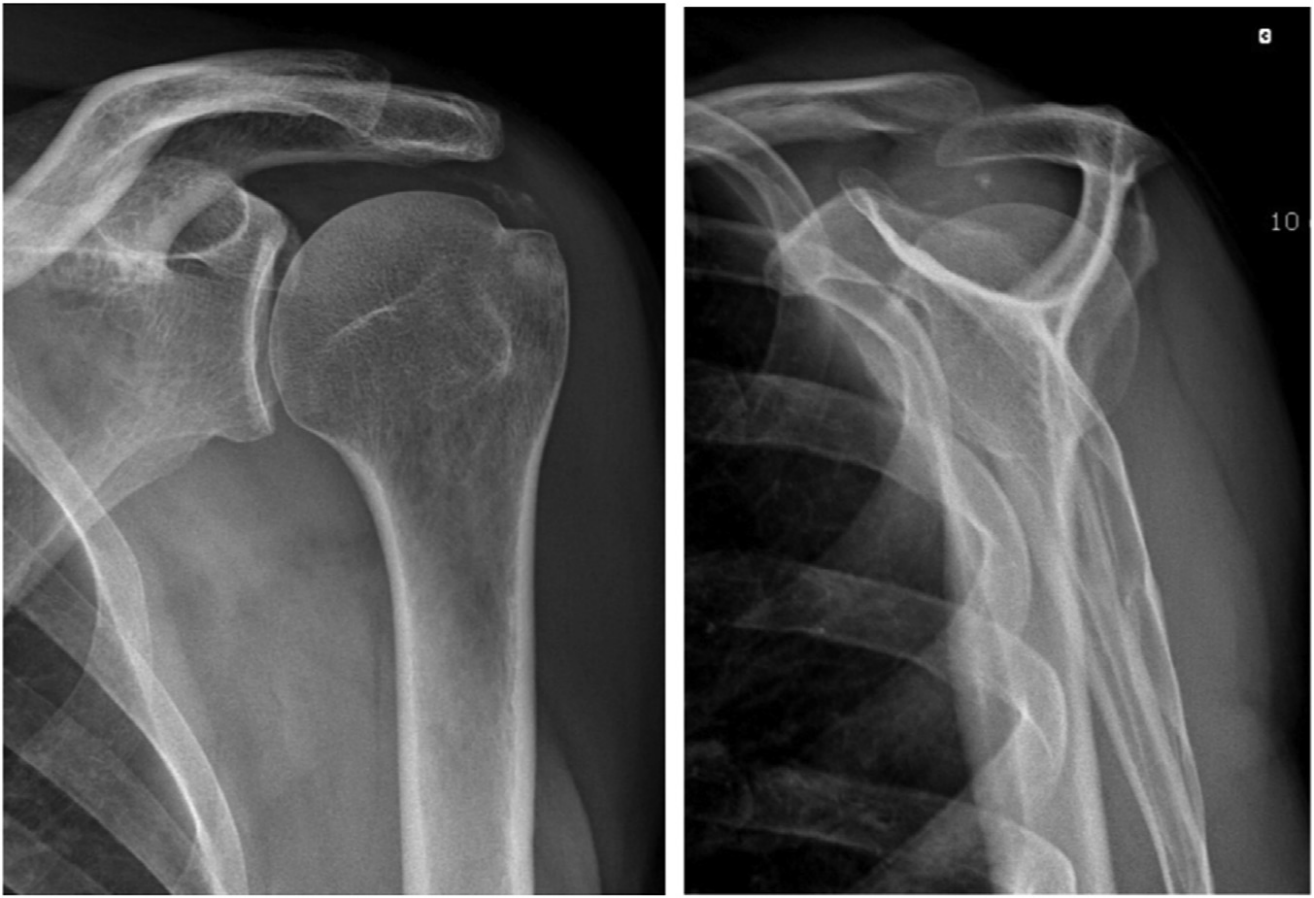
Anterior-posterior X-ray on the left and scapular Y profile of the patient on the right with an aspect of heterogeneous calcification of the supra spinatus tendon.

We report the case of a 62-year-old, right-handed man, who presented with a painful left shoulder that had persisted for 18 months, had recently worsened 2 months before and was more painful at night. Six months earlier the patient had undergone anterior-posterior (AP) and scapular Y profile X-rays that showed heterogeneous calcifications of the supraspinatus tendon (Fig. 1). He then resorted to painkillers and nonsteroidal anti-inflammatory drugs as only treatment. Following the failure of drug treatment alone, the patient was prescribed a nonoperative treatment based on shoulder physiotherapy and shock-wave therapy without infiltration.

**Figure 2:**
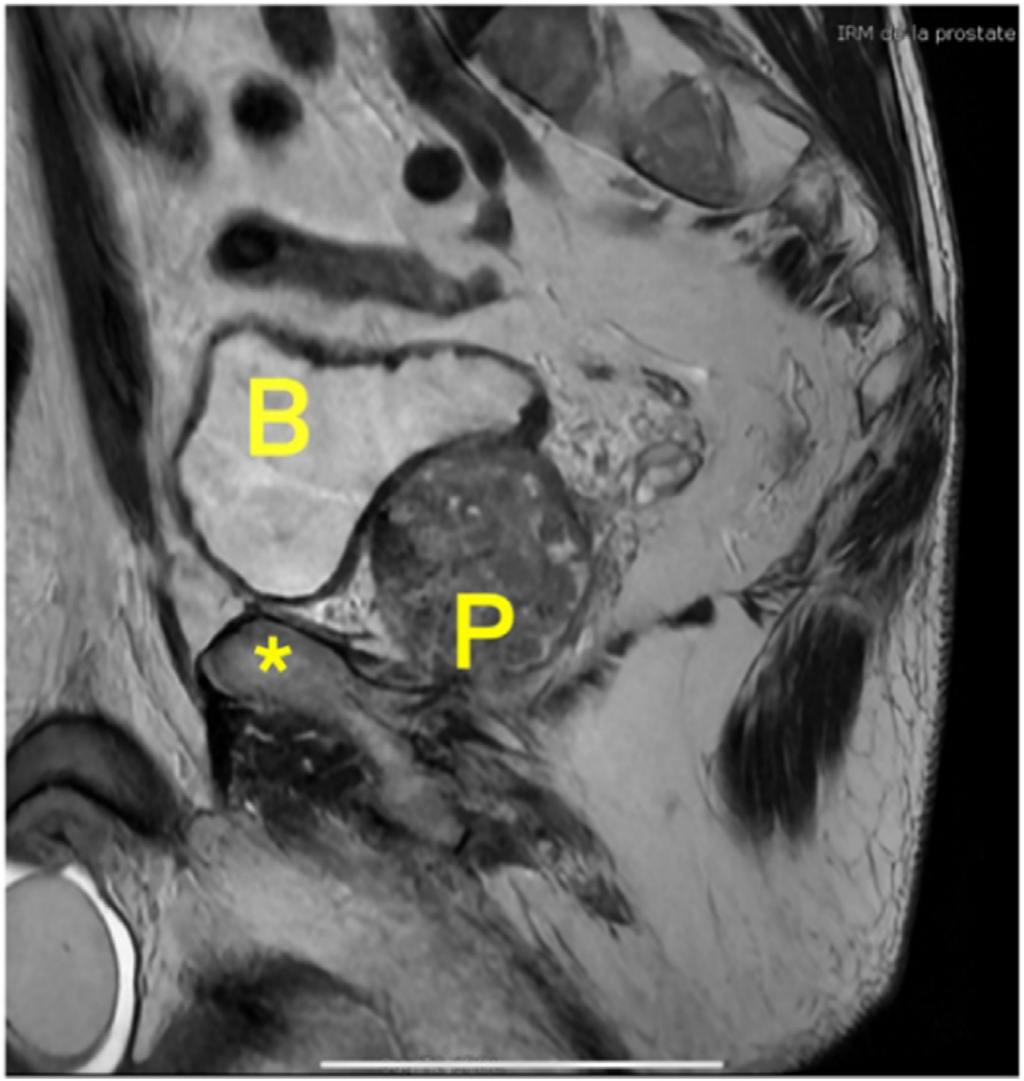
Sagittal T1 weighted MRI of the prostate showing 2 nodules with suspected capsular invasion in a potentially malignant lesion (∗ = Pubis). *B*, bladder; *P*, prostatis; *MRI*, magnetic resonance imaging.

During this nonoperative treatment and due to the age of the patient and the recent appearance of urination disorders, a serum prostate-specific antigen (PSA) assay was requested, revealing an abnormally high level. An MRI of the prostate was then prescribed, showing 2 nodules with suspected capsular invasion, suggesting a possible malignant lesion and justifying ultrasound-guided biopsy (Fig. 2).

**Figure 3:**
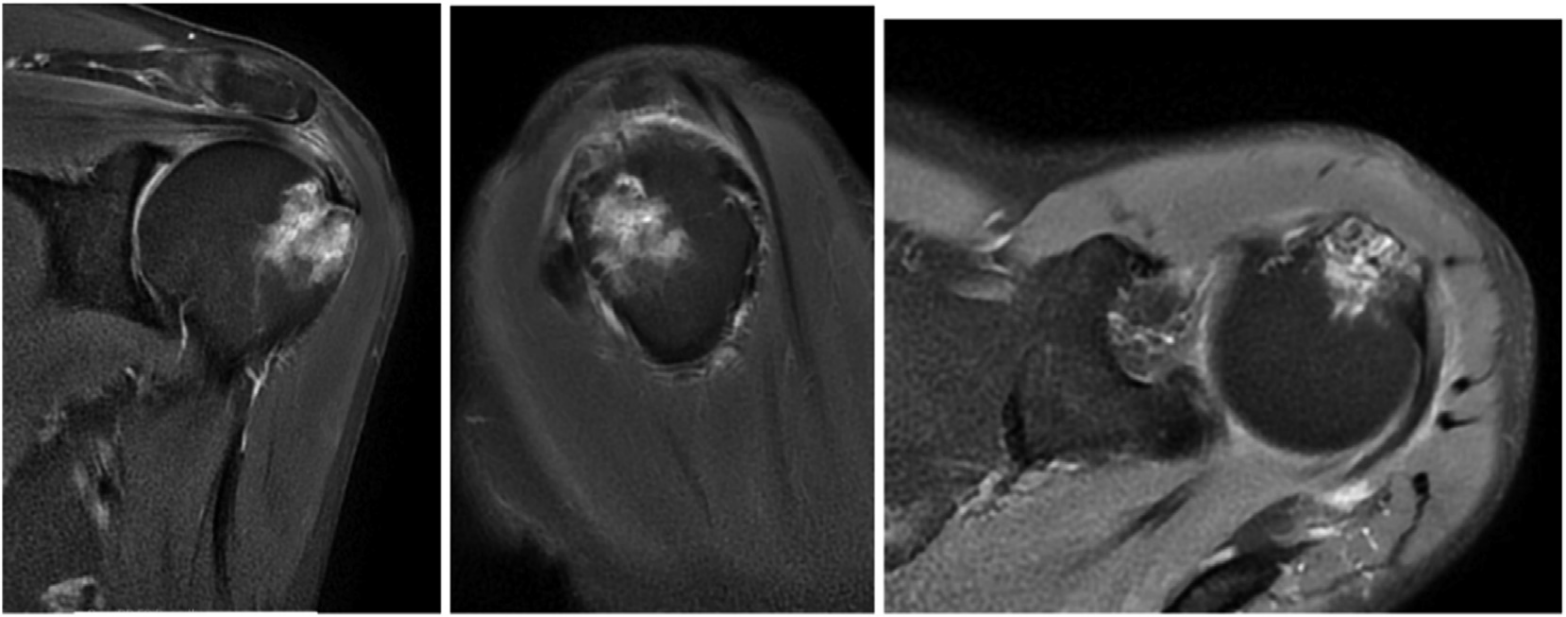
MRI with RhoFatsat sequences shows a significant intraosseous edema at the footprint of the supra tendinous tendon at the greater tubercule with irregularity and loss of cortical continuity. *MRI*, magnetic resonance imaging.

Due to the persistence of shoulder pain despite the nonoperative treatment and the recent suspicion of neoplastic disease, the imaging assessment has been completed with an MRI of the shoulder. This exam revealed on the RhoFat sat sequences a significant intraosseous edema next to the greater tubercle as well as an irregularity and loss of continuity of the cortex near the footprint with no calcium deposit clearly visible suggesting a secondary location of potential prostatic neoplasia (Fig. 3).

**Figure 4:**
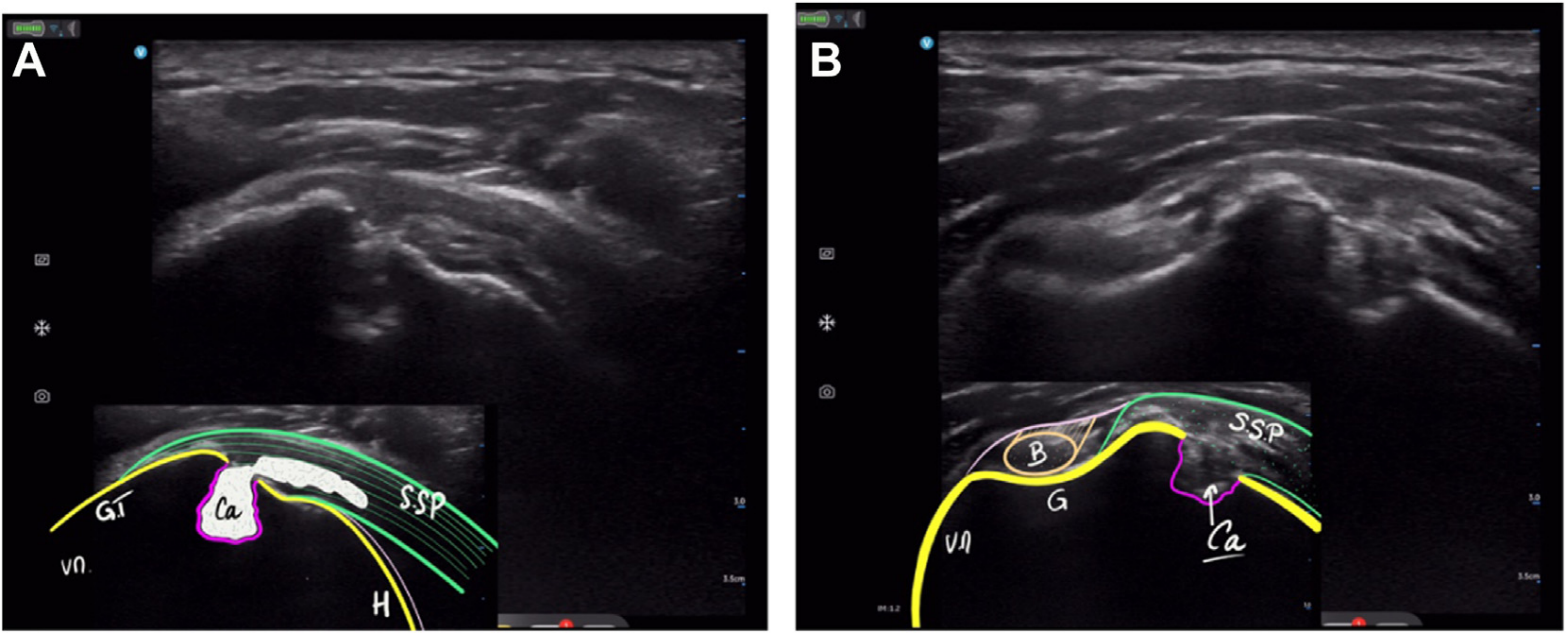
Ultrasound images performed by the surgeon during a consultation showing a calcium deposit in the supraspinatus tendon with intraosseous extension. (**A**) Longitudinal view. (**B**) Transverse view. *GT*, great tubercle; *H*, head; *Ca*, calcium deposits; *SSP*, supraspinatus tendon; *B*, biceps; *G*, gutter.

The patient was referred for an urgent orthopedic surgical consultation. Questioning clarified the existence of insomnia-causing pain for several weeks. The clinical examination revealed a rotator cuff tendinopathy, with signs of subacromial impingement and no restriction of range of motion. The surgeon performed an ultrasound examination during this consultation (Fig. 4), which confirmed the presence of persistent calcium deposits in the posterior part of the supraspinatus tendon. The continuity of the superior cortex near the footprint was also well visualized, as well as what appeared to be a second topographical variation of the calcification, which was intraosseous. No rotator cuff lesions were revealed.

**Figure 5:**
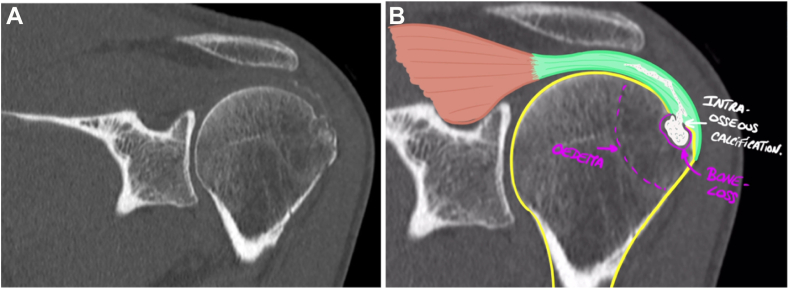
(**A**) Coronal CT scan showing intraosseous calcifications with bone edema and bone loss at the footprint of the supraspinatus tendon confirming the intraosseous migration of the calcification. The absence of extensive osteolysis is confirmed. (**B**) Drawing on the CT scan showing the supraspinatus tendon, intraosseous extension of calcium deposits, bone loss, and intraosseous edema. *CT*, computed tomography.

An urgent CT scan of the shoulder was prescribed, confirming the presence of an intraosseous calcium cluster at the great tubercle, and eliminating extensive osteolysis (Fig. 5).

The results of this examination excluded the hypothesis of humeral metastases and confirmed a rare diagnosis of intraosseous migration of a rotator cuff calcification. Due to the failure of the previous nonoperative treatment, the patient was offered surgery to remove the calcifications under arthroscopy with intraoperative ultrasound guidance.

### Surgical procedure

**Figure 6:**
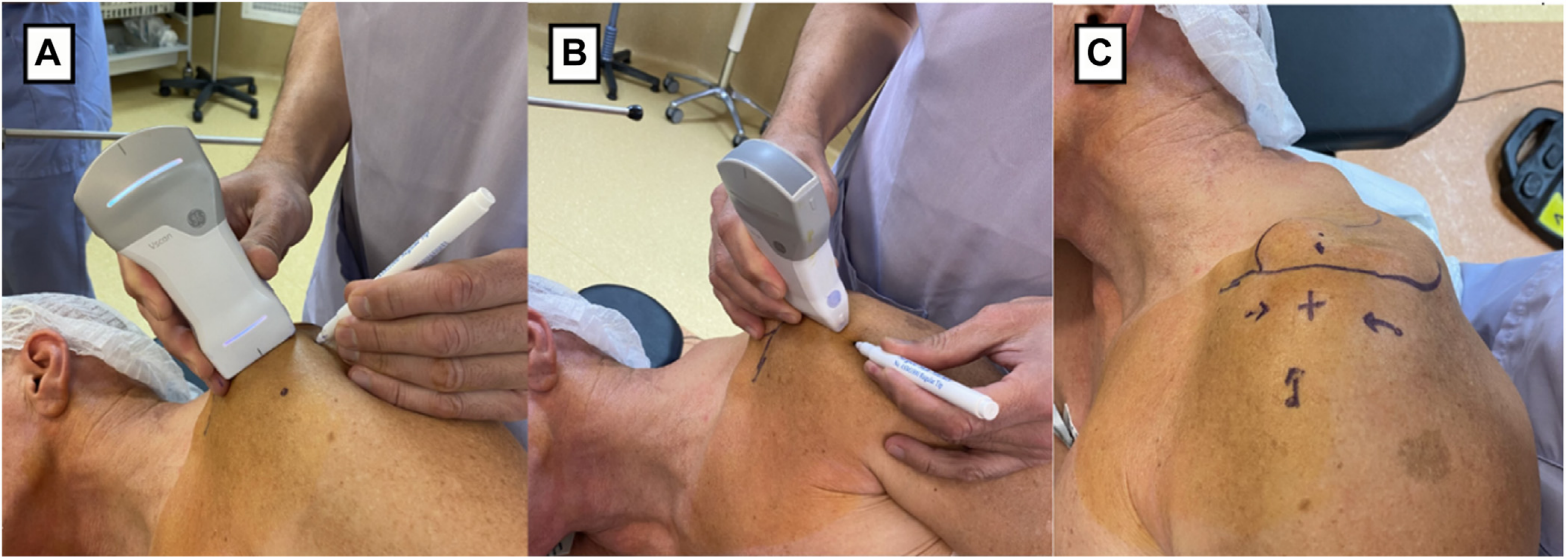
Preoperative checking just before aseptic skin disinfection of the good visualization of the calcifying tendinopathy on the patient in the future operative position. (**A**) Shows the longitudinal method and (**B**) the transversal method. (**C**) Illustrates the final aspect of the preoperative localization of the calcification.

The surgery was performed under general anesthesia and interscalene block with patient in the lateral decubitus. The A-USGN technique was performed step by step as initially described for intratendinous forms.^16^ Before skin disinfection the surgeon confirmed that both calcium topographies were clearly visible on ultrasound (Vscan Air; GE HealthCare, Chicago, IL, USA) with the patient in position for surgery (Fig. 6). A limited bursectomy was first performed to visualize the entire superficial part of the rotator cuff at the suspected location of the calcification. We identified intra tendinous calcification, causing the superficial face of the supraspinatus to bulge.

**Figure 7:**
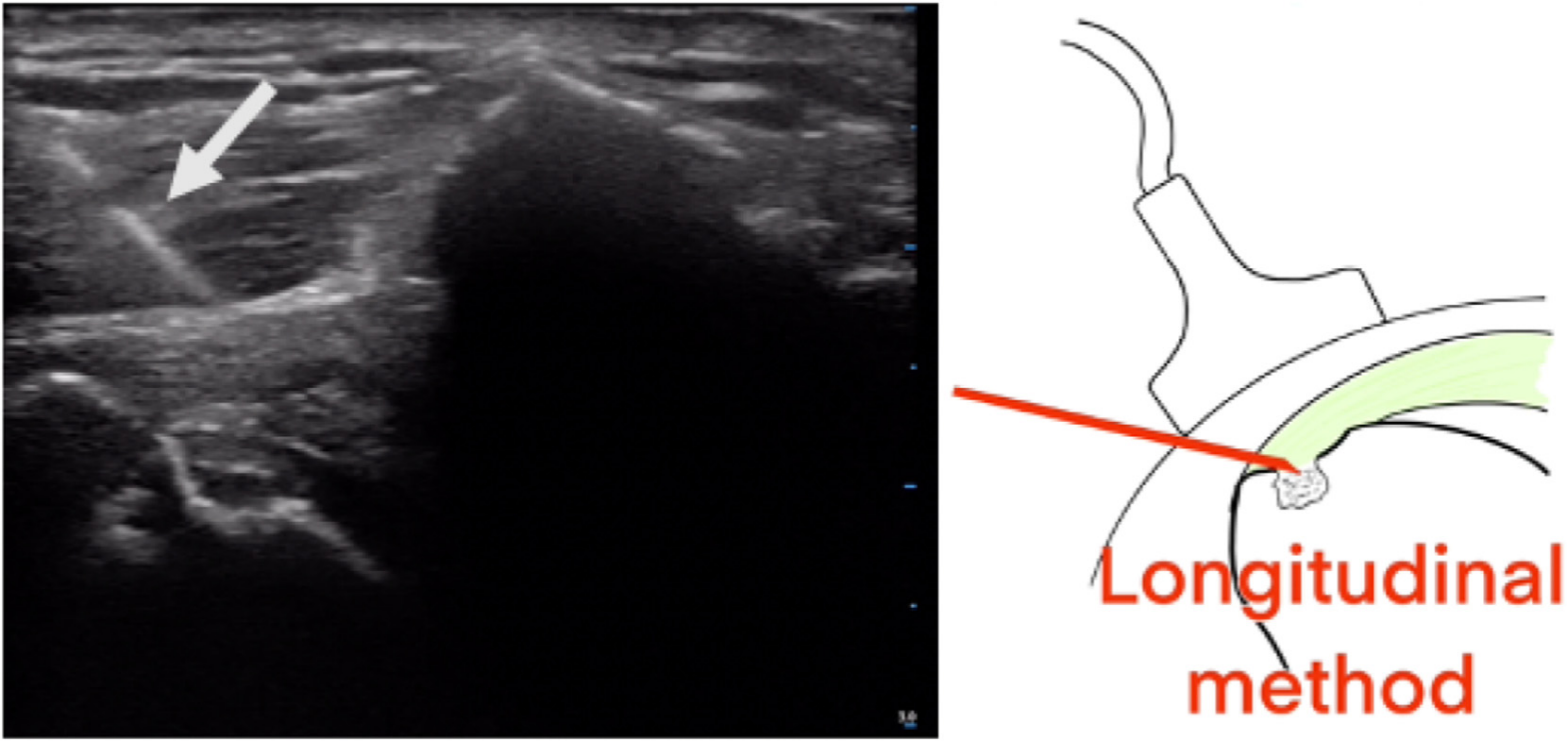
Use of the longitudinal method demonstrate an impossibility to well position the 24G hyperechoic needle inside the intraosseous calcification. The *arrow* identify the 24G hyperechoic needle that is used for the localization of the intraosseous calcification.

**Figure 8:**
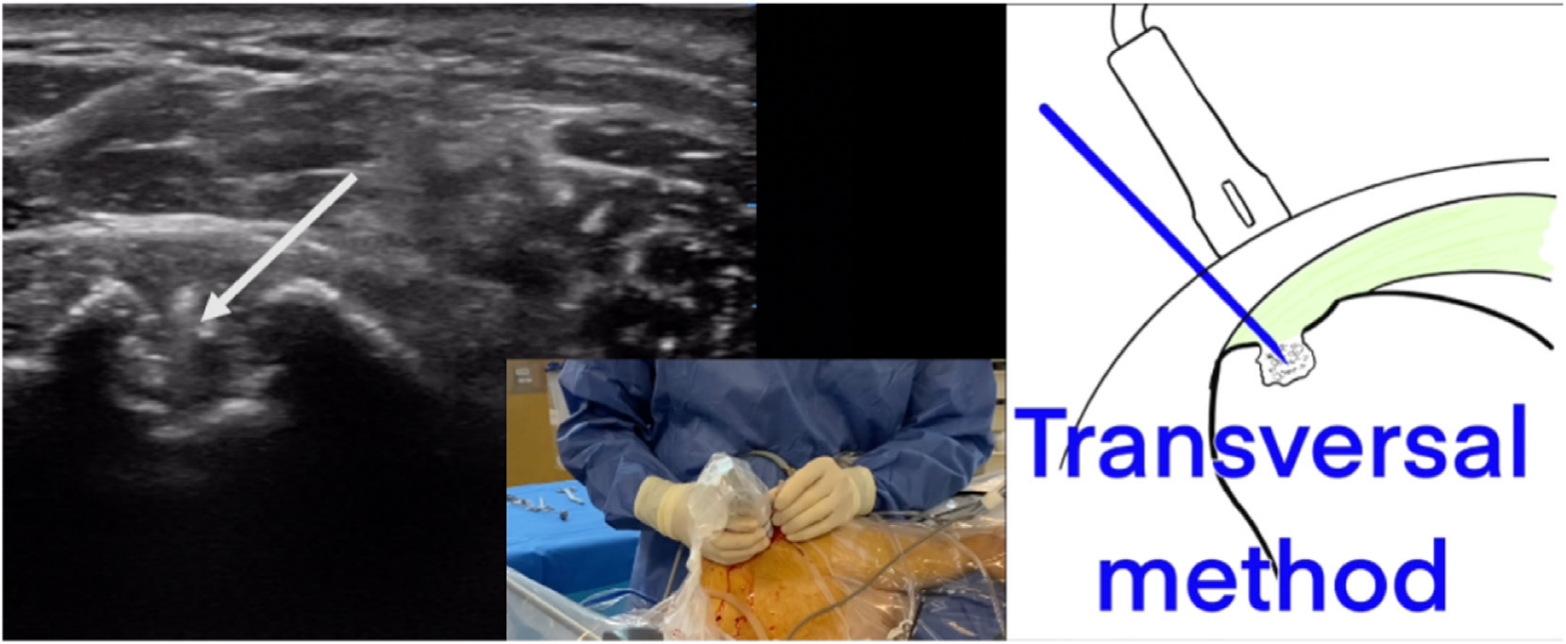
Echography images obtained using the transversal method which is perpendicular to the axis of the long head of the biceps and allows the good positioning of the 24G hyperechoic needle intraoperatively. The *arrow* identify the humeral intraosseous extension of the supraspinatus calcification.

The arthroscopic phase was then stopped to position a 24G hyperechoic needle inside the intraosseous calcification, initially with the needle parallel to the long axis of the probe (Fig. 7). However, insertion at this angle made it impossible to position the needle. Finally, the operator successfully positioned the needle transversely, which means perpendicular to the long axis of the probe, until he finally visualized the tip of the needle entering the bone defect (Fig. 8).

**Figure 9:**
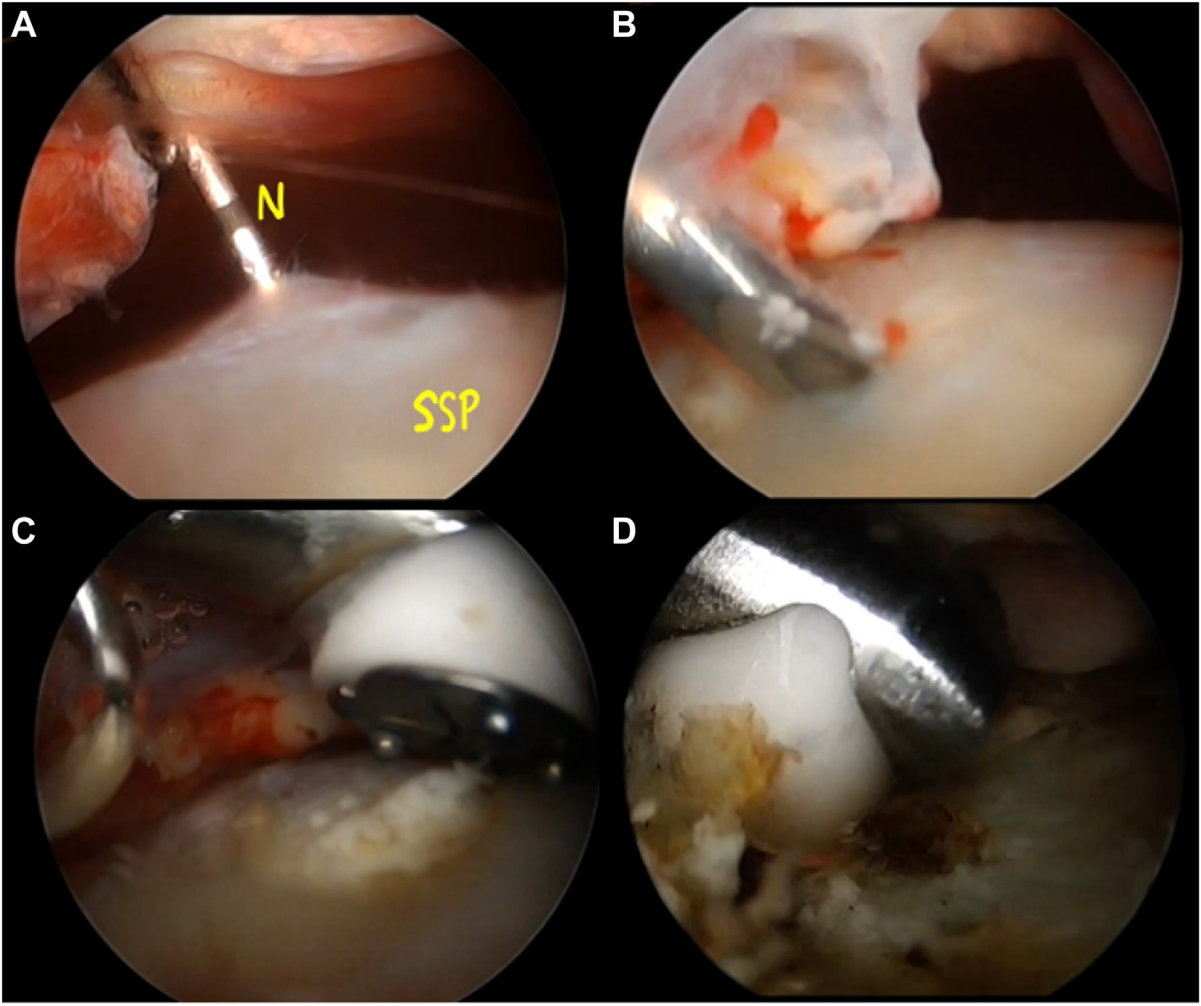
Arthroscopic bursal view of the opening of the supraspinatus tendon at the location of the 24G hyperechoic needle (**A**). The needle is first found in the subacromial space after the bursectomy (**B**). The needle is then used to initiate the removal of the calcification and facilitate the future use of the electrocoagulation probe (**C**). Then the superficial bursal side of the supraspinatus tendon is opened using the electrocoagulation probe (**D**). *N*, needle; *SSP*, supraspinatus.

The third stage consisted of repeating the arthroscopy and visualizing the point of needle penetration. The operator opened the superficial part of the tendon to resect the intratendinous topography (Fig. 9).

**Figure 10:**
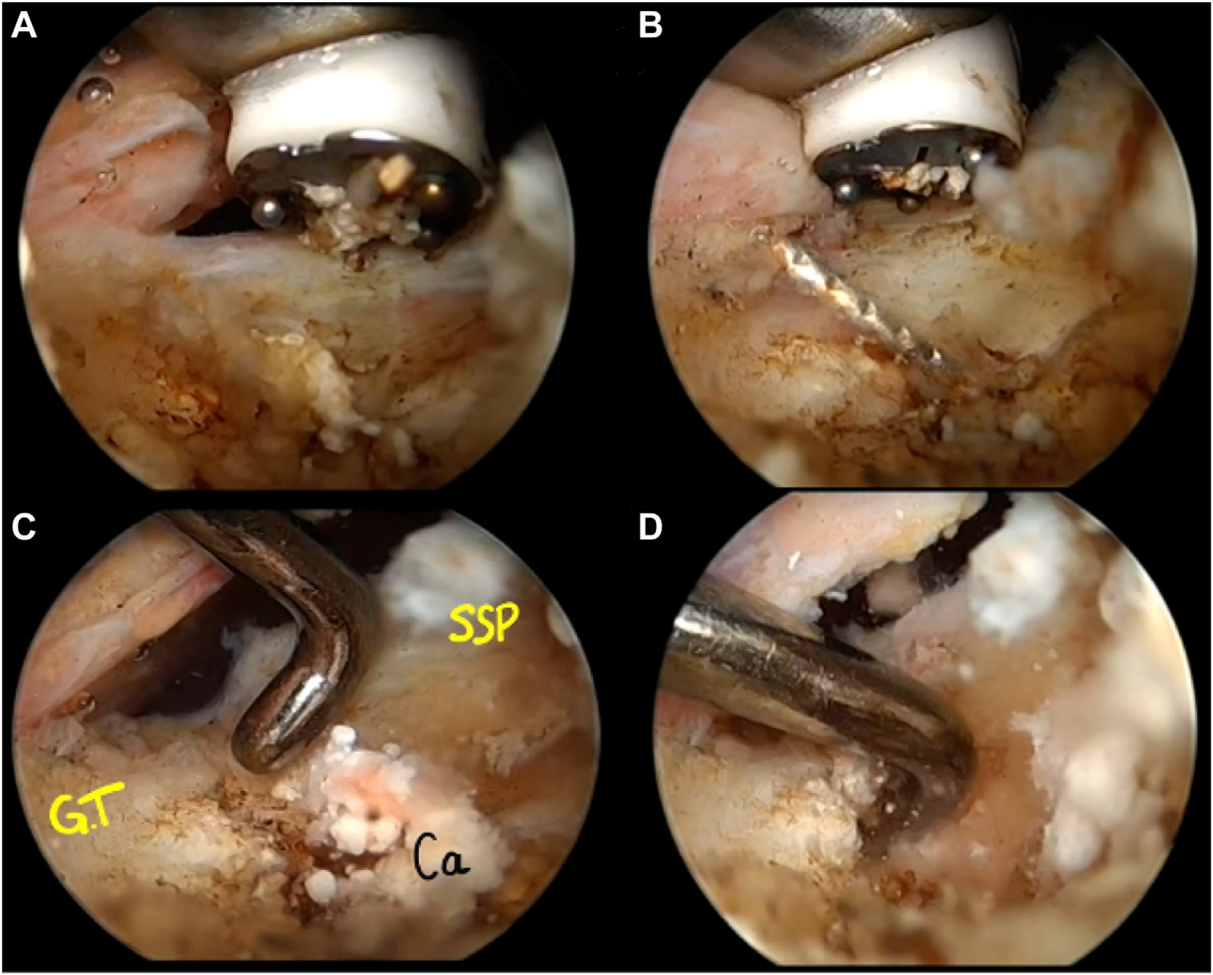
Arthroscopic bursal view of the opening of the minimal resection of the supraspinatus tendon until the cortical bone of the greater tuberosity (**A**). The needle is first localized (**B**) and then, using alternatively the probe and the electrocoagulation probe the intraosseous extension of the calcification can be localized (**C**) and progressively removed (**D**). *GT*, greater tuberosity; *Ca*, calcium deposit; *SSP*, supraspinatus.

**Figure 11:**
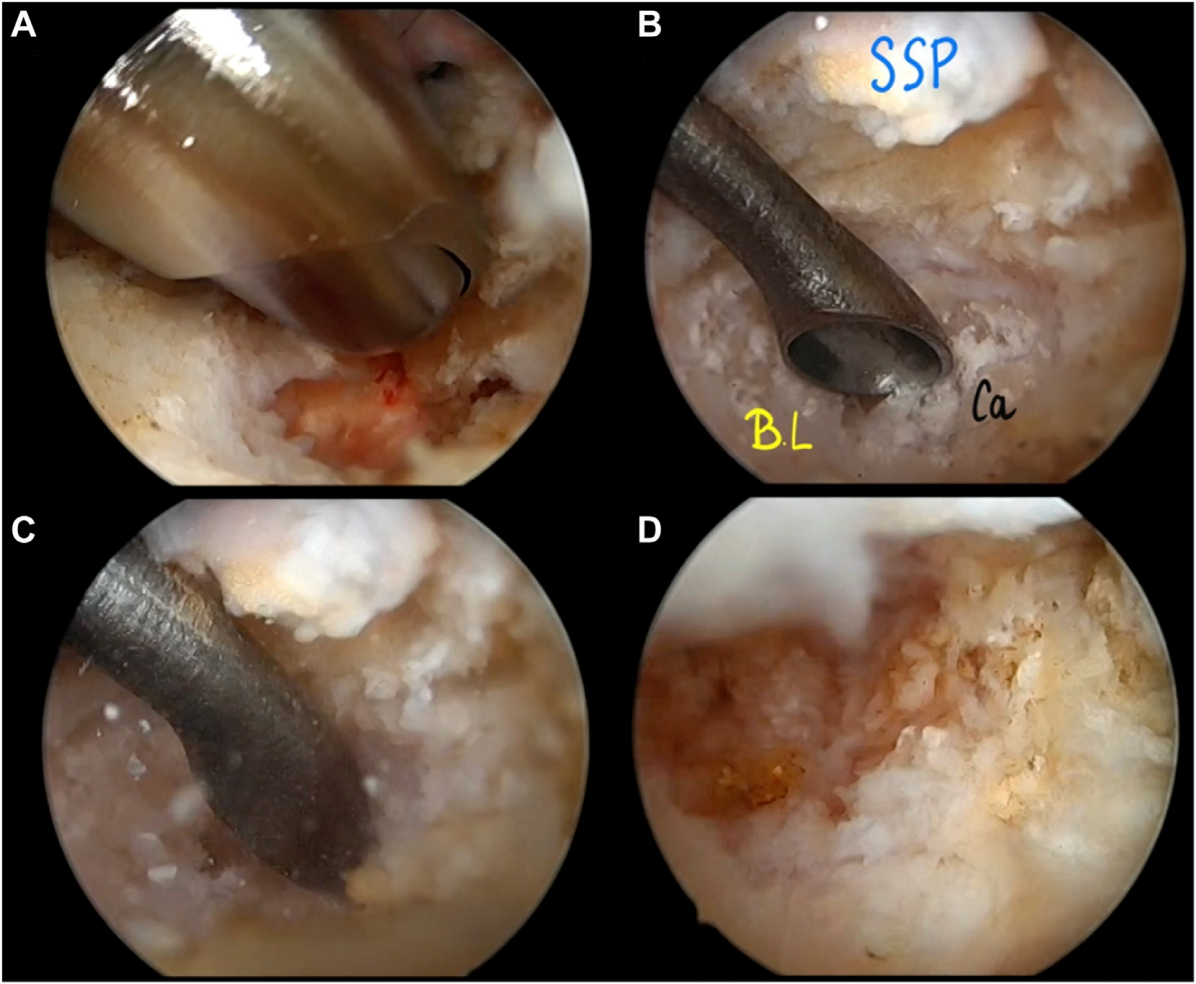
Arthroscopic bursal view of resection of the intraosseous extension of the calcification using of the probe, the shaver (**A**) and if necessary, a curette (**B** and **C**). Full resection of the calcification must be visualized at the end of the procedure through a portal located in front of it (**D**). *SSP*, supraspinatus; *BL*, bone lesion; *Ca*, calcium deposit.

During the fourth step, the supraspinatus tendon was incised where it was penetrated by the tip of the needle with minimal resection until the cortical plane of the greater tubercle was visible (Fig. 10). The calcification was then identified and carefully removed using the palpation hook and the shaver. Resection was incomplete, thus curettage was performed (Fig. 11). No tendon suturing was performed. All pre and intraoperative procedures are presented in Video 1.

### Postoperative recovery

The postoperative management included wearing of a resting splint for 2 weeks, and a self-rehabilitation protocol with exercises to be performed by the patient 3 times a day with no need for a physical therapist.

There was a postoperative follow-up visit 6 weeks after surgery. The patient had no pain and near complete recovery of range of motion. The patient also underwent a prostate biopsy which excluded any prostate cancer.

## Discussion

Intraosseous migration of rotator cuff calcifications is rare, and the incidence has not yet been clearly defined.^23^ In 1969, Moseley reported the first observation after surgery.^3^ The most frequent site remains supraspinatus insertion at the greater tubercle (76% of cases)^14^^,^^27^ and several reports have been published by radiologists,^6^ also describing damage to other location as the pectoralis major tendon.^11^^,^^27^ Only a little correlation has been demonstrated between findings associated with subacromial impingement and calcifying tendinitis.^13^

All the authors insist on the risk of misdiagnosis on MRI and confusing this entity with a primary or secondary bone lesion or osteoarthritis,^9^^,^^11^^,^^18^ due to the sometimes-severe extent of the perilesional edema like in the present case. This may lead to the performance in some cases of primary bone biopsies which could have been avoided a posteriori. In some cases, there is a risk of performing bone biopsies that could be avoided.^9^^,^^14^ On the other hand, if understanding of this entity is improved, conservative treatment could be proposed in clinically well-tolerated forms and may result in regression and resorption in a few months.^8^^,^^27^ It is important to combine different imaging examinations, in particular a nonenhanced scanner.^27^ The use of dynamic resonance imaging may be useful for patients with symptomatic chronic calcifying tendinitis showing an inflammatory signal around the calcium deposit or in the rotator interval and axillary pouch.^22^

Certain authors^9^^,^^14^ consider ultrasound to be a difficult examination for the diagnosis of intraosseous migration. With experience and especially more knowledge, ultrasound can reveal continuity from the cortical bone to the greater tubercle in the area of the tendinous insertion of the rotator cuff.^6^ This must be clearly differentiated from an acoustic shadow cone generated by a hard intratendinous calcification. The existence of continuity with neighboring intratendinous deposits helps determine the diagnosis.^14^ In the present case presented, it was possible to visualize intraosseous calcium deposits by adjusting the position of the probe.

The use of ultrasound by the orthopedic surgeon has been shown to be effective in the diagnosis of painful pathologies of the shoulder^4^^,^^10^ in particular for CTs.^28^ It rapidly provides additional information to refine the diagnosis, which may then be confirmed by a CT scan. The most important goal of this examination is confirming continuity between the bone lesion and tendon deposits [B] and the presence of contact between bone tissue and deposits.^11^

Although the learning curve for surgeons in ultrasound diagnosis is facilitated by the cross-referencing of preoperative and intraoperative imaging data^19^ but requires prior specific training.^21^ Martinel et al have previously reported that the use of intraoperative ultrasound for arthroscopic rotator cuff calcifications removal enabled a more complete extraction and prevented localization failure despite a tolerable increase in operative time (22 minutes with ultrasound technique vs. 18 minutes without, *P* = .3).^16^

The indication of the ultrasound-guided needling technique in the treatment of intraosseous localizations remains controversial. Gwalani et al reports its use in a single case with a good results,^6^ while Klontzas et al reports on a sample of 10 cases that found this procedure less effective than in the pure intratendinous forms.^12^ The studies by Seyahi et al^23^ and Caliskan et al^2^ reported a retrospective series of 10 and 29 patients treated by complete arthroscopic resection and cuff suture, with functional results identical to those of the group with intratendinous calcification. Moreover, the rate of complications that occurred when determining the therapeutic indication were similar. These results support the use of primary surgical management for the treatment of symptomatic forms.

We consider that once the ultrasound technique is well mastered by the orthopedic surgeon it may be use almost systematically in order to always perform an incision of the tendon at the right position to minimize the iatrogenic consequences but its main advantage are for the intraosseous localizations and the less accessible localization such as subscapularis, infraspinatus, and pectoralis major tendons. Before training in ultrasound, our operating surgeon had to operate on 4 other patients with intraosseous deposits at the major tubercle; they were systematically hard and not soft calcifications, embedded in the trabecular bone, requiring resection with a curette and a shaver knife to be complete.

The arthroscopic ultrasound technique for the extraction of intratendinous calcifications is now described and validated, showing better precision and less aggressiveness towards the tendon.^16^^,^^21^ All the difficulty in case of intraosseous localization is that it is necessary to make an opening of the tendon covering it. Conventional needle search exposes to a risk of significant tendon disinsertion and possible rotator cuff repair after excision.^18^^,^^21^^,^^23^

This case suggests that the use of ultrasound during arthroscopic surgery can improve the safety of the procedure and rapidly locate and confirm deposits. Incision of the tendon can then be limited and a tendon suture is not necessary. The A-USGN technique, described for pure intratendinous forms, is fully transposable to intraosseous forms.^17^

After a learning curve, intraoperative use of ultrasound remains easier and more efficient than fluoroscopy, because it is compact, allows immediate 3-dimensional identification and remains nonirradiating for patients and operating room professionals.^16^^,^^21^

## Conclusion

CTs of the cuff with intraosseous localization at the greater tuberosity remain rare and not well understood. Ultrasound is a nonirradiating imaging examination that can help orthopedic surgeon identify this rare entity and exclude other diagnoses in difficult cases.

The main benefits of this technique are the following: intraoperative 3-dimensional analyze of rotator cuff tendons, the minimal size of ultrasound probe in surgical sterile working space, low iatrogenic rate with probe placed in a sterile cover, and small tendon opening. This technique can help surgeon for intraosseous calcifications but also with intratendinous one that are difficult to locate, such as subscapularis and infraspinatus tendon. We believe that its use can be proposed for all calcification excision surgeries. The use of ultrasound during surgery improves the safety of resection and curettage of deposits and is less invasive to tendons.

## Disclaimers:

Funding: No funding was disclosed by the authors.

Conflicts of interest: Vincent Martinel reports that he has served as a conceptor and consultant for SBM society. The other author, his immediate family, and any research foundation with which he is affiliated have not received any financial payments or other benefits from any commercial entity related to the subject of this article.

Patient consent: Obtained.

## Data availability

Data will be made available on request.

## Supplementary Data

Video 1 This is the case of a 62-year-old man with a painful left shoulder for 18 months with a first diagnosis of calcifying tendinitis with no improvement after conservative treatment. A secondary localization has also been suspected because of urination disorders, an abnormal PSA serum level and an anormal pelvic MRI 2 prostatic nodules with a doubt of capsular crossing. Shoulder pain has increased, and MRI of the shoulder revealed a significant intraosseous edema of the greater tubercule suggesting a metastatic lesion. After clinical examination, an ultrasound examination was performed by a senior surgeon. The continuity of the superior cortex near the footprint was visualized as the calcium deposit in the posterior part of the supraspinatus tendon. Ultrasonography showed a second intraosseous topography of the calcification with calcium deposit visible in a bone defect on the longitudinal view. The transversal view confirmed the cortical bone defect of the greater tuberosity with calcium deposit visible inside the bone loss and an extension in the posterior part of the supraspinatus tendon. Intraosseous calcium deposit is visualized on a CT scan with edema around the bone loss. The surgical procedure consisted in a combination of the shoulder arthroscopy and an ultrasound-guided needling. After a classical bursectomy, the superior part of the rotator cuff was visualized. The Arthroscopic ultrasound-guided needling technique has been initiated using a 24-gauged hyperechogenic needle inside the intraosseous calcification initially with the needle parallel of the long axis of the probe. This procedure may have failed due to the orientation of the bone loss with the longitudinal echographic view. The use of the transversal view perpendicular with the long axis of the probe helped for the good positioning of the needle in the bone loss. The needle was arthroscopically visualized through the posterior portal. The needling could be performed under arthroscopic control and the superficial part of the tendon was opened using the electrocautery probe to evacuate the posterior intratendinous topography. The intraosseus calcifying resection was performed after supraspinatus tendon opening thanks to the tip of the needle with a minimal resection until the cortical plane of the greater tubercule was visible. The intraosseous ossification was then identified and carefully removed using the palpation hook, the shaver, and a curetage. The suture of the cuff at the end of the procedure was not mandatory.
